# Ankle pain and orientation after high tibial osteotomy as a treatment of medial compartment knee osteoarthritis

**DOI:** 10.1051/sicotj/2025051

**Published:** 2025-09-30

**Authors:** Moustafa Elsayed, Ahmed Lotfy Saber Mohammed, Abdelrhaman Elsheikh, Mohammed Ali Ahmed

**Affiliations:** Department of Orthopaedics, Faculty of Medicine, Sohag University Sohag 82524 Egypt

**Keywords:** Knee Osteoarthritis, Ankle Pain, Orientation, High Tibial Osteotomy, Medial Compartment Osteoarthritis

## Abstract

*Background*: Ankle pain frequently occurs in patients with medial compartment knee osteoarthritis (OA), particularly in those with varus deformity. In these patients, an atypical alignment of the ankle joint line relative to the ground is often observed in the coronal plane. The purpose of this study was to evaluate changes in ankle pain and ankle joint orientation after high tibial osteotomy as a treatment of medial compartment knee OA. *Methods*: This prospective work was conducted on 100 patients, aged 40–55 years old, with symptomatic medial compartment knee OA associated with ankle pain, with a good range of motion and intact lateral compartment. All patients treated with high tibial osteotomy fixed by plate. Ankle pain was measured by visual analogue score (VAS) preoperatively and at 3 months, 6 months, 1 year, and 2 years postoperatively. The following parameters were assessed preoperatively and at 3 months postoperatively: the ankle joint line orientation (AJLO), medial proximal tibial angle (MPTA), and the hip-knee-ankle angle (HKA). *Results*: The ankle pain significantly improved postoperatively and at last follow-up after HTO; VAS significantly reduced from 5 (4–5) preoperatively to 2 (1–2) at last follow-up (*P* < 0.001). AJLO was substantially decreased from 9.58 ± 2.74° preoperative to 0.41 ± 1.88° postoperative (*P* < 0.001). MPTA increased significantly following surgery, from a preoperative value of 85.78 ± 1.84° to a postoperative value of 90.71 ± 1.58° (*P*  <  0.001). Similarly, HKA improved significantly from −7.73 ± 1.50° preoperatively to 2.43 ± 0.88° postoperatively (*P* <  0.001). A positive correlation was found between ankle pain improvement via VAS and changes in AJLO, MPTA, and HKA (*P* < 0.05). *Conclusion*: In patients with medial unicompartmental knee OA associated with ankle pain, both ankle pain and ankle joint orientation improved following high tibial osteotomy.

## Introduction

Knee osteoarthritis (OA) is a common disorder. About 13% of women and 10% of men over 60 years of age have symptomatic knee OA. Degenerative changes in the knee more frequently affect the entire joint, including medial, lateral, and patello-femoral compartments [1, 2].

Medial compartment OA is 5–10 times more common than lateral compartment OA. Individuals with medial compartment knee osteoarthritis frequently have varus malalignment, and the load-bearing and mechanical axis passes through the medial compartment [3].

Ankle pain is often present in patients with medial compartment knee osteoarthritis associated with varus malalignment due to compensatory changes in the ankle joint, which leads to abnormality in ankle joint line orientation in the coronal plane (excessive lateral tilt) relative to the ground. In these cases, an increased prevalence of ankle osteoarthritis has been reported [4].

Among the few treatment options described for symptomatic medial compartment osteoarthritis, high tibial osteotomy (HTO) remains a favourable treatment option [5]. The targets of HTO are to minimize knee pain by shifting weight-bearing loads to the uninvolved lateral compartment in varus knees and to postpone the need for total knee replacement by slowing or arresting the destruction of the medial compartment [6].

Several techniques have been reported for HTO. The opening wedge high tibial osteotomy has increased in popularity because of its simple technique, and the subsequent total knee replacement is easier in comparison to the closing wedge HTO [7].

However, changes in the proximal tibial geometry after HTO could theoretically affect the orientation of both knee and ankle joints, which are directly related to the osteotomy site. Ankle joint orientation becomes more parallel to the ground after HTO. Ankle symptoms were affected by coronal alignment changes of the ankle after HTO [8, 9].

To date, few studies have evaluated changes in ankle pain following HTO [4, 8, 9]. The purpose of this research was to assess changes in ankle pain and ankle joint line orientation following high tibial osteotomy as a treatment of medial compartment knee OA with varus malalignment.

## Materials and methods

This prospective study was conducted on 100 patients, between 40 and 55 years of age, with symptomatic medial unicompartment OA associated with ankle pain, good range of motion, and intact lateral compartment.

Criteria for exclusion were the presence of ankle arthritis, previous operation at the knee or ankle joint, any foot deformity, combined lateral and medial compartment knee OA, markedly reduced knee range of motion, ligamentous instability, advanced patellofemoral osteoarthritis, and rheumatoid arthritis. Our ethical committee approved the study, and written consent was obtained from every participant.

Every patient was subjected to complete history taking, clinical examinations, routine laboratory tests, and radiological investigations [Standing whole lower limb radiograph and magnetic resonance imaging (MRI)].

**Figure 1:**
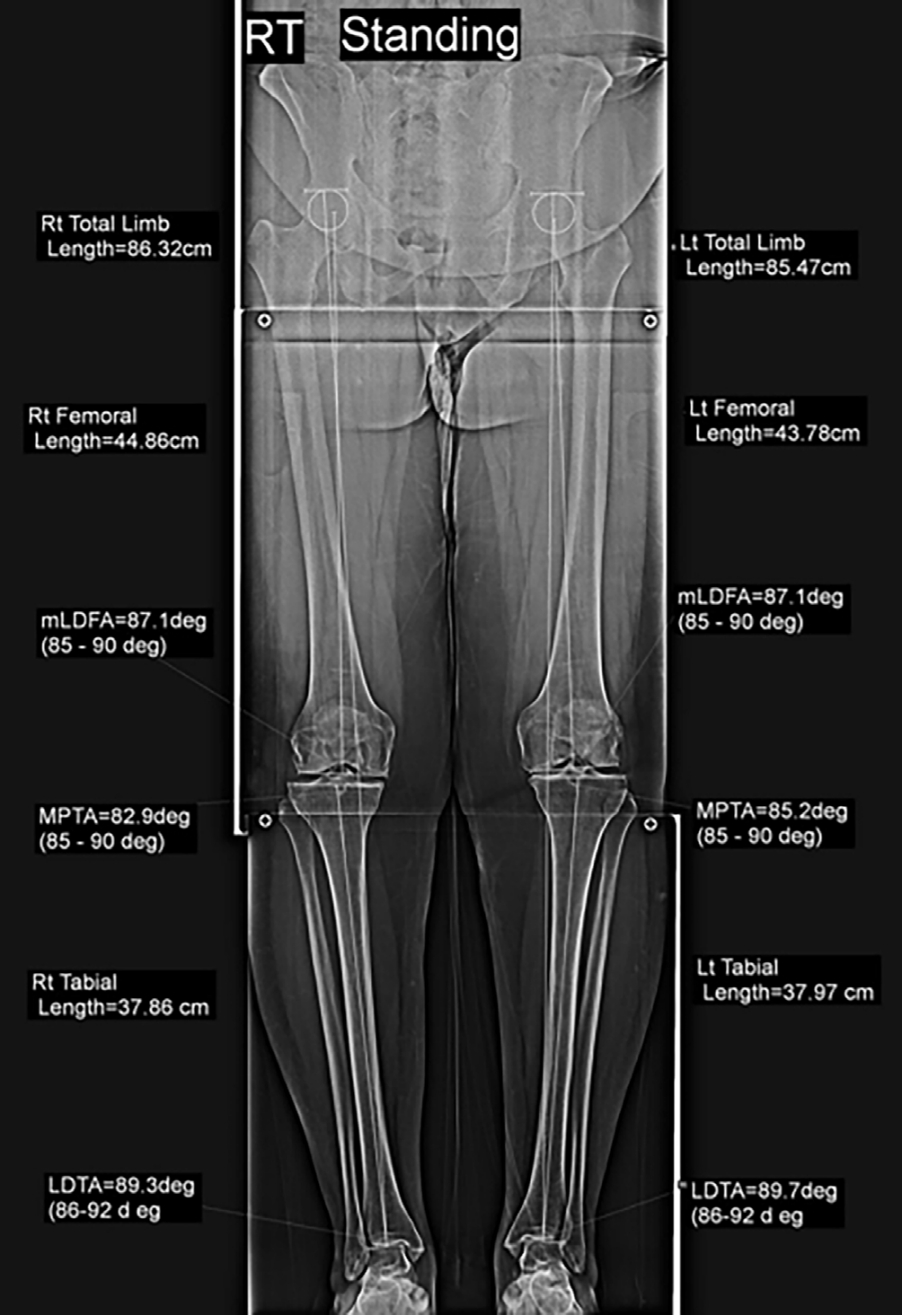
Preoperative planning using standing whole lower limb radiograph.

Full-length anteroposterior standing plain radiography of the lower extremities was conducted with the patella facing anteriorly. Preoperative planning for biplanar osteotomy using a standing whole lower limb radiograph to establish an accurate intraoperative correction angle (Figure 1), which is the angle at which the mechanical axis of the lower limb (Mikulicz line) intersects a point at 62.5% of the tibial plateau width [10].

### Surgical technique

The surgical procedure was done under spinal anaesthesia in the supine position. A preoperative broad-spectrum antibiotic was given to all patients. A tourniquet was applied to the operated limb. All surgeries were performed by the same surgical team of our knee unit.

At first, knee arthroscopy was done to address intra-articular joint abnormalities, including meniscal tears, cartilage lesions, removal of loose bodies, lavage, and synovectomy, as well as evaluation of the lateral compartment [11].

Then, a medial opening wedge osteotomy was performed under fluoroscopy as follows; a longitudinal incision was made in the medial aspect of the proximal tibia. The subcutaneous fat was dissected, and the pes anserinus tendons were identified and released from the medial tibial surface to expose the superficial part of the medial collateral ligament. The superficial medial collateral ligament was released from the bone by a sharp periosteal elevator.

**Figure 2:**
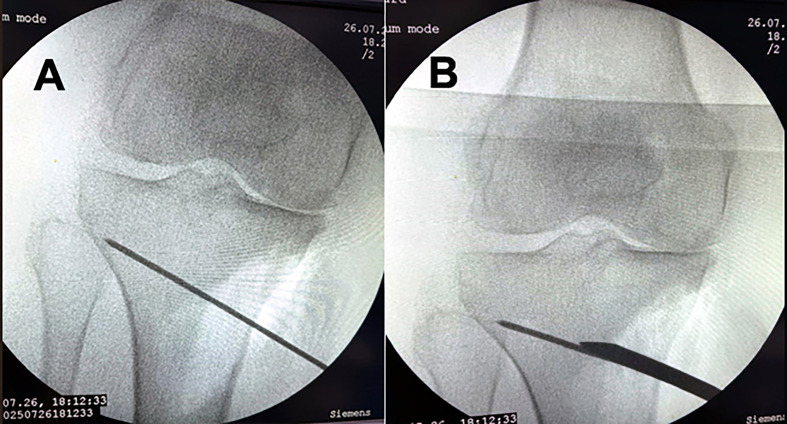
An intraoperative fluoroscopy picture; (A) A K-wire was inserted into the medial surface of the proximal tibia directed towards tip of the fibular head. (B) An osteotome was used to cut the bone in the direction of K-wire.

**Figure 3:**
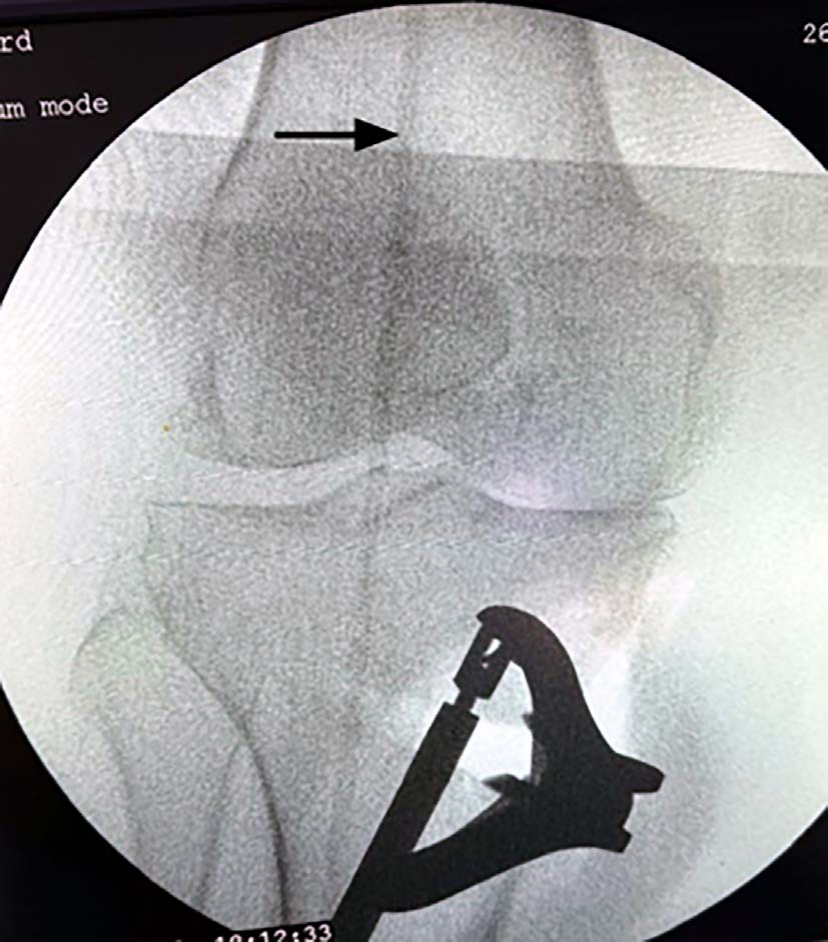
After opening the wedge, the alignment obtained was checked under fluoroscopy by a metallic wire (Black arrow).

**Figure 4:**
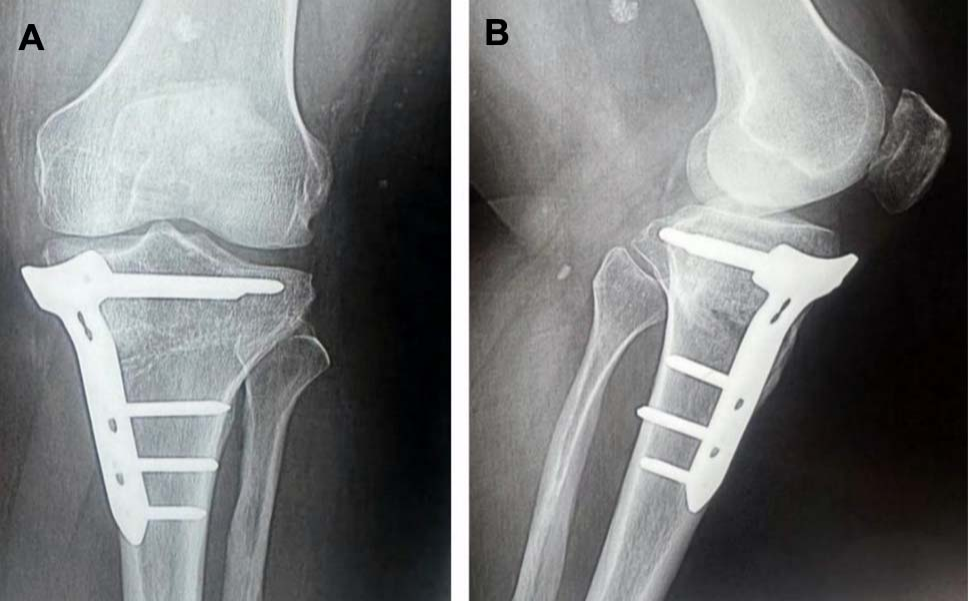
A 6-week postoperative knee X-ray (A) anteroposterior and (B) lateral views, showing healing of osteotomy site.

A 2.4-mm Kirschner guidewire was inserted into the medial surface of the tibia at the metaphyseal-diaphyseal junction just below the tibial tuberosity and directed proximally and laterally toward the tip of the fibular head. Upon verifying the proper placement of the wire, multiple drill holes were applied in the direction of the K-wire to the medial cortex to weaken the bone. The bone was cut using a sharp osteotome in the same direction as the K-wire guided by fluoroscopy while preserving the lateral cortex (Figure 2). The proximal part of the osteotomy was passed proximal to the tibial tuberosity. The wedge then opened according to the preoperative planning, and the alignment obtained was checked under fluoroscopy by a metallic wire (cable technique) (Figure 3) [9]. A locking plate, TomoFix Plate (Orthmed E), was used to fix the osteotomy. Finally, a drain was inserted, and closure of the wound was done in layers. Partial weight bearing was permitted after 2 weeks. Full weight bearing was permitted 6 weeks postoperatively after checking osteotomy healing by X-ray (Figure 4).

### Follow-up parameters

*Ankle pain was assessed by visual analogue scale (VAS):* This is shown as a 10 cm linear scale with the endpoints of “no pain” and “worst pain”. Ankle pain was evaluated preoperative and at 3 months, 6 months, 1 year, and 2 years postoperatively.

**Figure 5:**
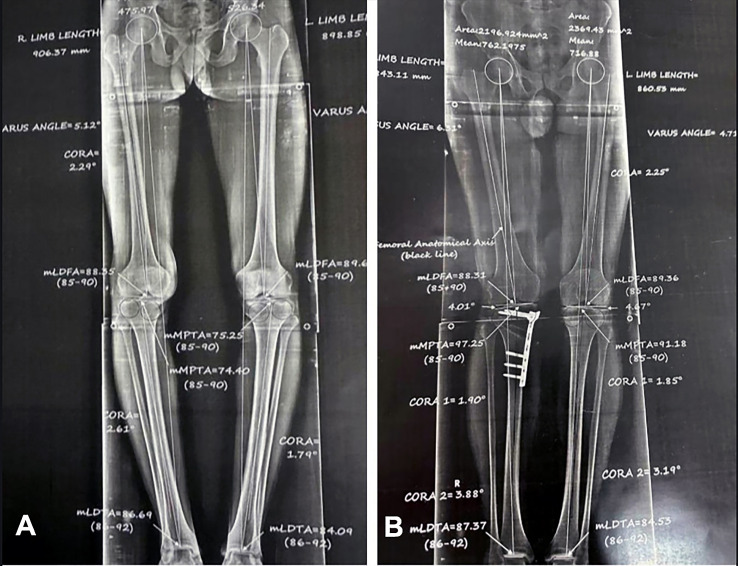
A standing whole lower limb radiograph (A) preoperative and (B) 3 months postoperative.

**Figure 6:**
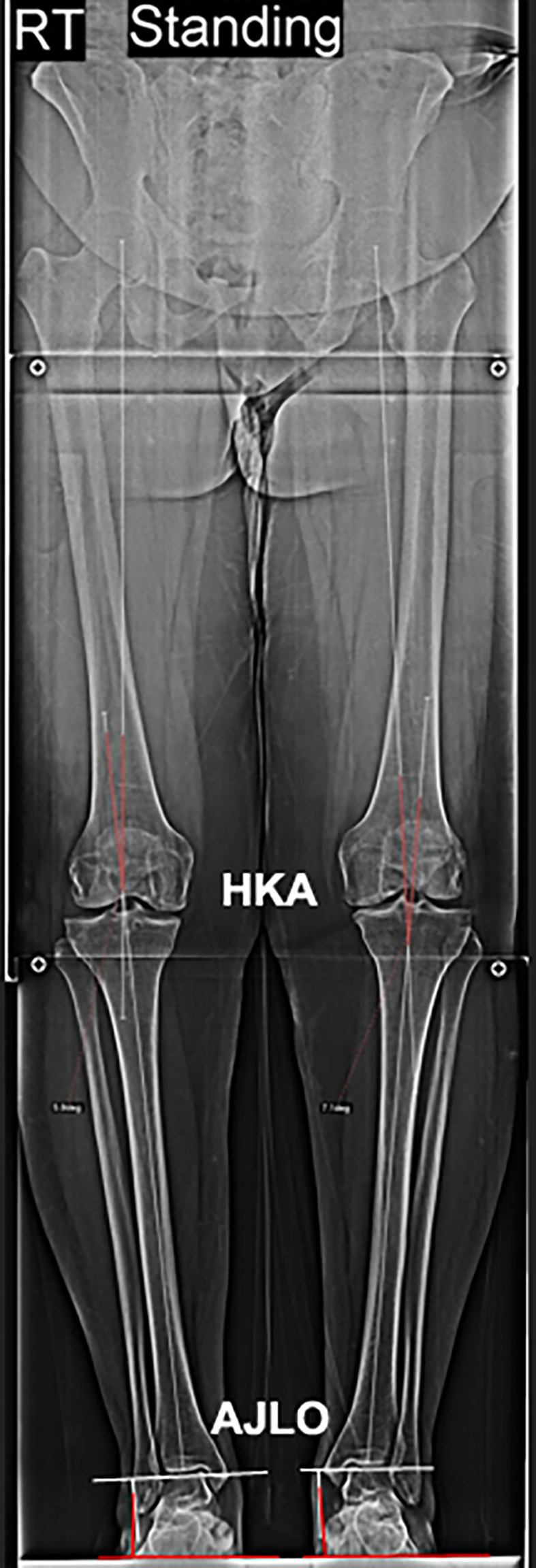
A standing whole lower limb radiograph showing Ankle joint line orientation (AJLO) and Hip-knee angle (HKA).

*Radiological parameters* were assessed blindly in a standing whole lower limb radiograph obtained 3 months postoperatively (Figure 5). It included:

- *Ankle joint line orientation (AJLO) relative to the ground angle*: It evaluates coronal alignment of ankle joint [12].
- The angle formed between the tangent to the dome of talus and the horizontal plane line on radiographs (Figure 6) is described as follows: a negative value is given when the tangent of the talus dome tilts medially in relation to the horizontal plane line.
- *Medial proximal tibial angle (MPTA)*: is the medial angle created between the tibial axis and the knee joint line of the tibia in the frontal plane (Figure 1) was measured preoperatively and after 3 months [12].
- *Hip-knee angle (HKA)*: To assess the coronal alignment of the knee and correction of the coronal plane. It is the angle formed when the mechanical axis of the femur is extended through the distal femur to form an angle with the tibial mechanical axis (Figure 6). It is given a negative value when in varus orientation and a positive value when in valgus [12].

### Statistical analysis

Statistical analysis was performed utilising SPSS v26 (IBM Inc., Chicago, IL, USA). The Shapiro-Wilks test and histograms were utilised to evaluate the normality of data distribution. Quantitative parametric data were displayed as mean and standard deviation (SD) and were contrasted by repeated measures ANOVA. Quantitative non-parametric data were displayed as median and interquartile range (IQR) and were contrasted by the Wilcoxon test. Qualitative parameters were expressed as frequency and percentage (%). A two-tailed *P*-value < 0.05 was considered statistically significant.

The sample size calculation was done by G*Power 3.1.9.2 (Universität Kiel, Germany). The sample size was based on the following considerations: 0.383 effect size, 95% confidence level, 95% power of the study, and five cases were added to overcome dropout. Therefore, we recruited 100 patients in this study.

## Results

This study included 100 patients, 47 (47%) men and 53 (53%) women. The mean age was 47.8 ± 7.56 years. The affected Side was right in 34 (34%) patients. Regarding MRI findings, 60 (60%) of patients had degenerative posterior horn medial meniscus tear, 30 (30%) of patients had knee joint effusion, 5 (5%) of patients had baker’s cyst, and 4 (4%) of patients had medial synovial plicae (Table 1).

**Table 1:** Demographic data and MRI findings of the patients.

		*N* = 100
Age (years)		47.8 ± 7.56
Sex	Male	47 (47%)
	Female	53 (53%)
Affected side of knee	Right	34 (34%)
	Left	66 (66%)
Degenerative posterior horn medial meniscus tear		60 (60%)
Knee joint effusion		31 (31%)
Baker’s cyst		5 (5%)
Medial synovial plicae		4 (4%)

Data are presented as mean ± SD or frequency (%). MRI: magnetic resonance imaging.

Ankle pain assessed by VAS was significantly improved postoperatively. Median VAS improved from 5 (IQR 4–5) preoperatively to 2 (IQR 1–2) at final follow-up (*P* < 0.001) (Table 2).

**Table 2:** Ankle pain assessed by VAS of the studied patients.

	Preoperative	Postoperative			
		After 3 months	After 6 months	After 1 year	After 2 years
Ankle pain assessed by VAS	5 (4–5)	4.5 (4–5)	3 (2–4)	2 (1–3)	2 (1–2)
*P*-value compared to postoperative		<0.001*	<0.001*	<0.001*	<0.001*

Data are presented as median (IQR). *Significant as *P*-value ≤ 0.05. VAS: visual analogue score.

AJLO was substantially decreased from 9.58 ± 2.74° preoperative to 0.41 ± 1.88° postoperative (*P* < 0.001). MPTA increased significantly following surgery, from a preoperative value of 85.78 ± 1.84° to a postoperative value of 90.71 ± 1.58° (*P* <  0.001). Similarly, HKA improved significantly from −7.73 ± 1.50° preoperatively to 2.43 ± 0.88° postoperatively (*P* <  0.001). Table 3: A positive correlation was found between ankle pain improvement via VAS and changes in AJLO, MPTA, and HKA (*P* < 0.05) (Table 4).

**Table 3:** Radiological parameters of the studied patients.

	Preoperative	Postoperative	Delta change	*P*
HKA (°)	−7.73 ± 1.5	2.43 ± 0.88	10.18 ± 1.68	<0.001*
MPTA (°)	85.78 ± 1.84	90.71 ± 1.58	4.95 ± 1.52	<0.001*
AJLO	9.58 ± 2.74	0.41 ± 1.88	−9.15 ± 3.30	<0.001*

Data are presented as mean ± SD. *Significant as *P*-value ≤ 0.05, HKA: Hip knee ankle angle, MPTA: Medial proximal tibial angle, AJLO: Ankle joint line orientation.

**Table 4:** Correlation between ankle pain (VAS) and delta change of radiological parameters.

		Ankle pain
HKA (°)	*R*	0.235
	*P*	**0.022**
MPTA (°)	*R*	0.243
	*P*	0.017*
AJLO	*R*	0.222
	*P*	0.030*

*r*: Pearson coefficient, *Significant as *P*-value ≤ 0.05, HKA: Hip knee ankle angle, MPTA: Medial proximal tibial angle, AJLO: Ankle joint line orientation.

Three cases suffered from superficial infection, which improved with repeated dressing and broad-spectrum antibiotics.

## Discussion

Medial compartment knee OA with varus malalignment is frequently associated with ankle pain due to compensatory changes occurring in ankle joint line orientation, which may progress to ankle OA if neglected [4]. Few studies in the literature discussed the effect of HTO or total knee replacement on ankle joint orientation and subsequently ankle pain.

In the present study, Ankle pain assessed by VAS was significantly improved from 5 (4–5) preoperatively to 2 (1–2) at the last follow-up (*P* < 0.001). this improvement in ankle pain was supported by improvement in radiological parameters; AJLO was significantly decreased from 9.58 ± 2.74° preoperative to 0.41 ± 1.88° postoperative (*P* < 0.001), which means that the ankle joint line became more parallel to the ground. This was associated with the accuracy of our correction assessed by MPTA, which increased significantly following surgery, from a preoperative value of 85.78 ± 1.84° to a postoperative value of 90.71 ± 1.58° (*P* <  0.001), which means improvement in knee varus. Also, global limb alignment was assessed by HKA, which improved significantly from −7.73 ± 1.50° (varus deformity) preoperatively to 2.43 ± 0.88° (valgus) postoperatively (*P* <  0.001).

Unlike our results, Kim et al. [4] demonstrated that, despite the patient-reported outcome measurements for the knee joint showing improvement, ankle pain worsened following HTO in individuals with ankle OA. Kim et al. [4] included 130 patients with medial compartment knee OA associated with ankle OA. They used different radiological parameters than we used to assess ankle alignment. They concluded that ankle pain worsened after HTO in these patients due to inadequate compensatory change in the hindfoot. While in our study we excluded patients with ankle OA, this may explain the difference in results in respect to this study.

Prior clinical reports indicated that the rectification of severe genu varum abnormalities following TKA or HTO correlates with a decline in post-operative ankle functionality. Graef et al. [13] showed that adjustments of ≥14.5° might elevate the incidence of ankle complaints by 15.6 times. Chang et al. [14] indicated that ankle discomfort was exacerbated following TKA in individuals with concurrent ankle OA.

Regarding this study, AJLO was significantly lower postoperatively compared to preoperativlye. In accordance with our findings, Abo-Tahra *et al.* [8] measured AJLO pre and after HTO in patients with medial compartment knee OA and stated that AJLO was significantly lower postoperatively compared to preoperatively. They concluded that HTO changed the relative AJLO, which became more parallel to the ground; this phenomenon may improve the biomechanics of the ankle joint by permitting more even distribution of the weight on the ankle joint. Although these findings are similar, unlike our study, they did not evaluate ankle pain clinically.

In the same line, Kim et al. [9] found that AJLO relative to the ground was significantly lower postoperatively compared to preoperatively, and the change in the HKA angle had a significant correlation with that of AJLO in cases with genu varum deformity corrected after knee arthroplasty or HTO.

Our results showed that a positive association existed between the change of MPTA, HKA, and AJLO. In accordance with our findings, Mohamed Abo-Tahra et al. [8] illustrated that a change of the Mechanical tibiofemoral angle (mTFA) has a statistically positive correlation (*P* = 0.01) with AJLO. This is because the valgus compensation of the ankle joint that occurs in the varus knee would be neutralized after correction of varus by valgus HTO.

Regarding MRI findings, 60 (60%) patients had degenerative posterior horn medial meniscus tear, 30 (30%) patients had knee joint effusion, 5 (5%) patients had baker’s cyst, and 4 (4%) patients had medial synovial plicae. Supporting our results, Culvenor et al. [15] demonstrated that 44 studies, including 3,761 knees from 2,817 individuals, indicated a pooled prevalence estimate of meniscal tears at 10%. Research including participants with a mean age of less than 40 years showed a pooled prevalence of 4% (ranging from 2% to 7%), whereas those aged 40 years and beyond had a prevalence of 19% (spanning from 13% to 26%). Forty-two studies (4322 knees from 3446 people) revealed the prevalence of cartilage abnormalities, yielding an overall pooled prevalence estimate of 24%, whereas the prevalence of effusion/effusion-synovitis varied from 0% to 92% across 21 studies. Landsmeer et al. [6] reported that the prevalence was 70% for cartilage defects, 66% for meniscal abnormalities, and 52% for meniscal extrusions.

Limitations of this work involved a small number of patients, the absence of long-term follow-up, functional scores, or objective gait analysis, and being a single-centre study, which may result in findings that differ from those obtained elsewhere. The study also lacked a control group to compare the findings.

Despite these limitations, this study is among the few studies evaluating the effect of HTO on the ankle joint clinically and radiologically. However, further studies using different functional scoring systems and different radiological parameters are needed.

## Conclusions

Ankle pain and ankle joint line orientation significantly improved after high tibial osteotomy in patients with medial unicompartment knee OA with varus deformity.

## Data Availability

Data associated with this article cannot be disclosed due to legal reasons.
